# Half-Elemental Diet Shifts the Human Intestinal Bacterial Compositions and Metabolites: A Pilot Study with Healthy Individuals

**DOI:** 10.1155/2020/7086939

**Published:** 2020-08-06

**Authors:** Jun Miyoshi, Daisuke Saito, Mio Nakamura, Miki Miura, Tatsuya Mitsui, Toru Kudo, Shinnosuke Murakami, Minoru Matsuura, Tadakazu Hisamatsu

**Affiliations:** ^1^Department of Gastroenterology and Hepatology, Kyorin University School of Medicine, 6-20-2 Shinkawa, Mitaka-shi, Tokyo, Japan 1818611; ^2^Nutrition Department, Kyorin University Hospital, 6-20-2 Shinkawa, Mitaka-shi, Tokyo, Japan 1818611; ^3^Metabologenomics, Inc., 246-2 Kakuganji-Mizukami, Tsuruoka-shi, Yamagata, Japan 9970052

## Abstract

**Methods:**

This prospective study included four healthy volunteers. The subjects continued their dietary habits for 2 weeks after the registration of the study and then started half-ED replacing 900 kcal of the regular diet with ED (time point 1, T1). The subjects continued half-ED for 2 weeks (T2). After the withdrawal of ED, subjects resumed their original dietary habits for 2 weeks (T3). Fecal samples were collected from all subjects at all time points, T1-3. Fecal DNA and metabolites were extracted from the samples. We performed 16S rRNA gene amplicon sequencing and metabolomic analysis to examine the bacterial compositions and intestinal metabolites.

**Results:**

There were differences in the gut bacterial compositions and metabolites at each time point as well as overtime changing patterns between subjects. Several bacteria and metabolites including short-chain fatty acids and bile acids altered significantly across the subjects. The bacterial membership and intestinal metabolites at T3 were different from T1 in all subjects.

**Conclusions:**

Half-ED shifts the gut bacterial compositions and metabolites. The changes varied with each individual, while some microbes and metabolites change commonly across individuals. The impact of half-ED may persist even after the withdrawal. This trial is registered with UMIN ID: 000031920.

## 1. Introduction

Inflammatory bowel diseases (IBD) are chronic intestinal and systemic disorders. The incidence and prevalence of IBD are increasing globally. The pathogenesis of IBD remains unclear. Crohn's disease (CD) is one of the major clinical phenotypes of IBD. CD causes progressive destructive damage to the gastrointestinal (GI) tract [1]. Accumulated GI damage by chronic inflammation leads to irreversible structural and functional changes. Therefore, it is crucial to achieve and maintain remission quickly and sustainably. Today's therapeutic goal for CD is the endoscopic remission (mucosal healing) beyond the clinical remission. Various studies demonstrated that earlier intervention can improve the clinical outcomes of CD patients [2]; however, optimizing the CD therapy remains an important challenge. For this point, enteral nutrition (EN) is an “old” but “new” therapeutic option. Voitk et al. first reported the efficacy of EN for IBD in 1973 [3], and several studies examined the efficacy of EN for pediatric and adult CD [4]. The guideline on clinical nutrition in IBD by the European Society for Clinical Nutrition and Metabolism (ESPEN) [5] and the consensus guideline for pediatric CD by the European Society of Pediatric Gastroenterology, Hepatology and Nutrition (ESPGHAN) and the European Crohn's and Colitis Organization (ECCO) [6] state that exclusive EN (EEN) can be the first-line induction therapy for CD patients especially those who are still growing and partial EN may be a therapeutic option together with other medications for the maintenance therapy. A recent meta-analysis concluded that, for the induction of remission in CD, EN should be considered in patients who can tolerate this therapy or when steroid side effects need to be avoided [7]. Furthermore, in this biologic era, it is a crucial finding that concomitant Elental®, an elemental diet (ED), improves the anti-TNF*α* antibody therapy outcome by reducing the loss of response [8, 9]. ED is one of the types of EN that contains individual amino acids, sugars (mono- or polysaccharides), and low fat. The previous studies demonstrated the efficacy of ≥900 kcal/day of ED [8, 9]. In addition, this dosage of ED shows the preventive effect against the postoperative CD recurrence as well [10]. Considering the patients' tolerance, for CD patients with the indication, introducing ED from 3 packs/day (900 kcal/day) is common in Japan. Thus, with its safety, EN started to be used decades ago and still plays an important role in CD treatment according to the evolution of therapeutic strategies. However, the mechanisms of how EN/ED exerts its clinical efficacy remain undetermined. Diet is a strong driver for shifting the gut microbiota [11–14], and the interactions between the gut microbiota and the host influence the host immune system [15, 16]. Several studies showed that the exclusive EN influences the gut microbiota in pediatric CD patients [17]. It was also suggested that there is a difference in bacterial compositions between adult CD patients with/without ED (>1200 kcal/day for >6 months) [18]. Here, we hypothesized that ED alters the gut microbiota to the anti-inflammatory condition leading to clinical efficacy. There is considerable interindividual variability in the gut microbiome [19], and the GI inflammation can be confounding factors when assessing the impact of ED on the human gut environment [20]. In addition, investigating not only microbial structure but also metabolism could provide more insights into the host-microbe interactions [21]. Therefore, in the present study, we recruited healthy volunteers and tracked them over time examining not only the gut bacterial compositions but also the intestinal metabolites to investigate the impact of EN on the gut microbiota as the functional community.

## 2. Materials and Methods

### 2.1. Study Design and Ethics

Four healthy volunteers were recruited in the present study. Four individuals applied for the study, and the informed consent was obtained from them (4 subjects; K1-4, 1 female and 3 males, 34-51 years old) (Table 1). None of the subjects had any past medical history. They had not taken probiotics, antibiotics, or other medications for at least 6 months before the study as well as during the study. After the registration of 4 subjects, a registered dietician had interviews with subjects to ask their dietary habits (T0). The subjects were instructed not to change their dietary habits for 2 weeks. After 2 weeks (T1; week 0), the subject started 900 kcal/day of elemental diet (ED; Elental®). The nutritional components of Elental® are shown in Table 2. At this point, the subjects were instructed to reduce dietary calorie intake by 900 kcal/day so that the daily calorie intake did not change in total. The subject continued the elemental diet for 2 weeks, and their adherence to ED was tracked. After this 2-week intervention, the subjects resumed their original dietary habit (T2; week 2). The observation period was completed 2 weeks later (T3; week 4). All subjects submitted the records of their meal contents, and the registered dietician analyzed their nutritional intakes (total calories and the ratio of protein : fat : carbohydrate (PFC ratio)) at all time points, T1-T3. Fecal samples and blood samples were collected from the subjects at the time points, T1-3 (Figure S1). Fecal samples were used for further analyses in the present study. This study was approved by the Institutional Review Board of Kyorin University School of Medicine (IRB No. 720), registered in University hospital Medical Information Network (UMIN) Clinical Trial Registry (UMIN ID: 000031920), and performed in accordance with the principles of the Declaration of Helsinki.

### 2.2. Fecal DNA Extraction and 16S rRNA Gene Amplicon Sequencing Analysis

Fecal DNA extraction and 16S rRNA gene analysis were performed as previously described [22]. Briefly, the V1-2 region of the bacterial 16S rRNA gene was amplified using universal primers 27F-mod (5′-AGRGTTTGATYMTGGCTCAG-3′) and 338R (5′-TGCTGCCTCCCGTAGGAGT-3′) with Tks Gflex DNA Polymerase (Takara Bio Inc., Shiga, Japan) and sequenced by using MiSeq (Illumina, CA, USA) in paired-end mode. The obtained paired-end sequences were merged using vsearch (version 1.9.3) (options: --fastq_maxee 9.0 -fastq_truncqual 7 -fastq_maxdiffs 300 -fastq_maxmergelen 450 -fastq_minmergelen 250) [23]. After removing of PhiX and primer sequences using cutadapt (options: -O 13 -m 50 -M 450 -q 0; -e option is not used in cut 5′ primer, but used 0.3 in cut 3′ primer) and bowtie2 (version 2.3.4.3) with default settings, the sequences were filtered by read quality (25 or higher) using an in-house script. The high-quality sequences were mapped onto SILVA SSU Ref NR 99 reference sequences using bowtie2 (version 2.3.4.3) (option: --no-hd -no-sq -no-unal -I 280 -X 400 -fr -no-discordant -phred33 -D 15 -R 10 -N 0 -L 22 -I S,1,1.15 -q) to assign to operational taxonomic units (OTUs) [24, 25]. For each fecal specimen, random subsamples of 10,000 sequences were employed for the following microbiome analysis.

### 2.3. Fecal Metabolite Extraction and CE-TOFMS Measurement

Fecal metabolites were extracted after adding an internal standard following previously reported procedures [26] with a minor modification: final resuspension volume was changed to 50 *μ*l of Milli-Q water. The metabolome analysis was performed with CE-TOFMS. Annotation tables were produced from a measurement of standard compounds and were aligned with the datasets according to similar *m*/*z* value and normalized migration time. Based on the peak area of the internal standard, the relative areas of each metabolite were calculated. For specific metabolites, the absolute abundances were calculated based on the relative peak areas and concentrations of the standard compounds corresponding to the metabolites.

### 2.4. Statistics

Statistical analyses were performed with R software (version 3.3.3) (https://www.R-project.org/). To compare bacterial relative abundances and relative areas of metabolites between the three timepoints, the Conover test was conducted as a post hoc test following the Friedman test using an R library “PMCMR” (https://CRAN.R-project.org/package=PMCMR) with default setting (*p*.adjust.method = “none”). Spearman's rank correlation coefficient was analyzed to examine the correlations between fecal bacteria and metabolites. To evaluate the false discovery rate, *q* values were calculated from *p* values of the Friedman test and Spearman's rank correlation analysis, respectively, by “qvalue” function of an R library “qvalue” (https://github.com/StoreyLab/qvalue). In these analyses, bacteria showing insignificant levels (less than 0.001 relative abundance) were excluded. Statistical significance was assumed when *p* < 0.05.

## 3. Results

### 3.1. Elemental Diet Induces Persistent Shifts of the Gut Microbial Structure Varying with Each Individual

**Figure 1:**
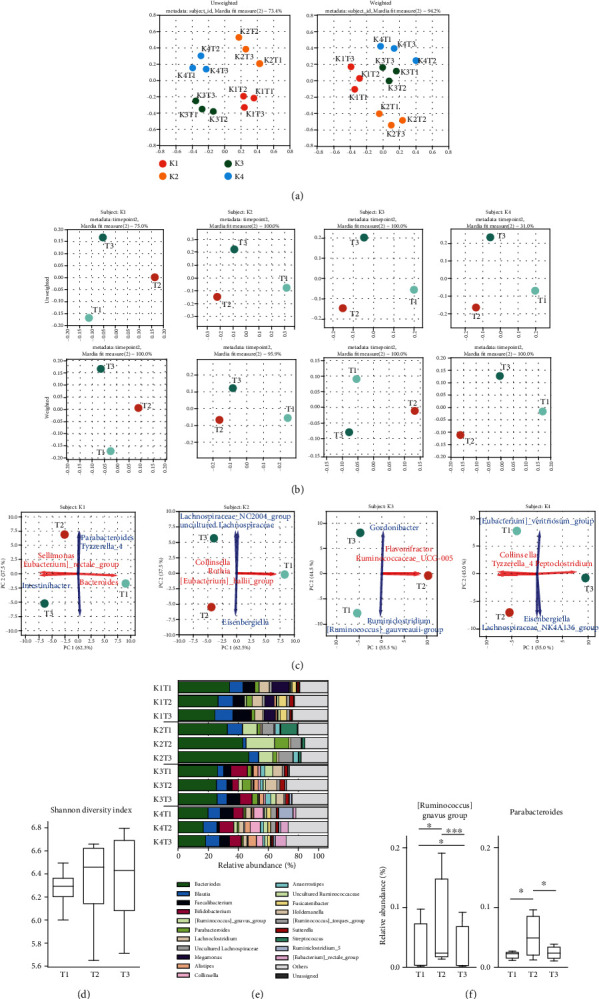
Elemental diet induces persistent alterations of the gut microbial membership varying with each individual. (a) Principal coordinate analysis (PCoA) plots of both unweighted and weighted UniFrac distances for operational taxonomical units (OTUs) demonstrated that bacterial compositions were different between subjects at all time points. (b) PCoA plots for each subject demonstrated that the bacterial compositions were different between time points. (c) Principal component analysis (PCA) plots at the genus level demonstrated that different genera were affected by ED between subjects. The top3 loadings of PC1 and PC2 are shown with red arrows and blue arrows, respectively. (d) The Shannon diversity index did not change significantly between time points. (e) The relative abundances at the genus level in the fecal samples from the subjects K1-4 at T1-3. (f) The relative abundances of *[Ruminococcus]_gnavus_group* and *Parabacteroides* changed over time.

The background of each subject and nutrition at each time point (T0-3) is shown in Table 1. The calorie intake of subject K1 at T3 decreased due to professional pressure, not due to health problems. The gut bacterial structure was assessed based on the 16S rRNA gene amplicon sequencing, resulted in 8,941 OTUs (Table S1). The changes of the gut microbiome membership were investigated by comparing the bacterial relative abundances between time points (T1-3) at several taxonomic ranks, i.e., OTU, genus, family, and phylum, respectively. Principal coordinate analysis (PCoA) plots of both unweighted and weighted UniFrac distances for all subjects demonstrated interindividual differences in bacterial compositions at all time points (Figure 1(a)). Unweighted UniFrac distances describe the OTUs existing in samples, while weighted UniFrac distances consider the proportions of those OTUs. In addition to the difference at T1, the pattern of bacterial shifts over time varied among subjects. PCoA plots for each subject demonstrated the bacterial membership at T3 (2 weeks after cessation of ED) was still different from that at T1 (Figure 1(b)). Principal component analysis (PCA) plots at the genus level with the top3 loadings of PC1 (red arrows) and PC2 (blue arrows) demonstrated that different genera were affected by ED between subjects (Figure 1(c)). The Shannon diversity index calculated from the OTU levels did not significantly change between time points (Figure 1(d)).  Figure 1(e) represents the relative abundances at the genus level of all of the 12 samples (K1-4 at T1-3, respectively). For genera of *[Ruminococcus]_gnavus_group* and *Parabacteroides* (longitudinal changes with *p* < 0.0183 and *p* < 0.0498, respectively), multiple comparisons between time points were performed. *[Ruminococcus]_gnavus_group* and *Parabacteroides* significantly increased at 2 weeks after starting ED (T2) compared to the onset (T1) but decreased at 2 weeks after the withdrawal of ED (T3) (Figure 1(f)). The other statistical results on longitudinal changes in OTUs and taxonomies (*p* < 1.00) are shown in Table S2.

### 3.2. Elemental Diet Alters the Intestinal Metabolite Profiles over Time in Each Individual

**Figure 2:**
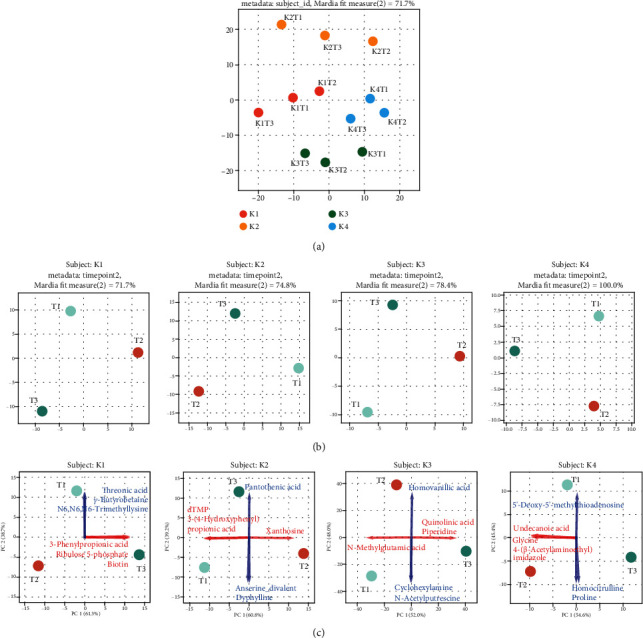
Elemental diet induces persistent changes of the intestinal metabolite profiles varying with each individual. (a) Principal coordinate analysis (PCoA) plots of Euclidean distances demonstrated that metabolite profiles were different between subjects at all time points. (b) PCoA plots for each subject demonstrated metabolite profiles were different between time points. (c) Principal component analysis (PCA) plots demonstrated that different genera were affected by ED between subjects. The top3 loadings of PC1 and PC2 are shown with red arrows and blue arrows, respectively.

**Figure 3:**
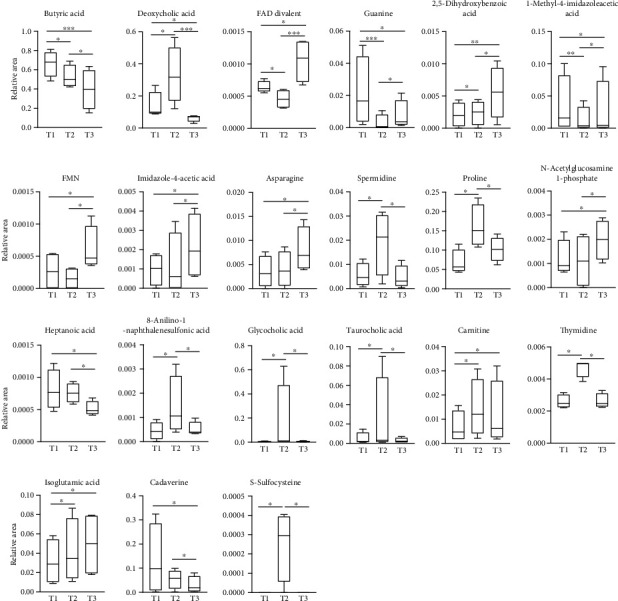
Changing patterns of metabolites with significant differences over time. Among 316 metabolites analyzed in the present study, 21 metabolites showed significant differences between the time points.

The relative areas of 316 fecal metabolites were obtained in the present study. The chronological changes of metabolites were analyzed based on their relative areas. PCoA plots of Euclidean distances of all of the 12 samples (K1-4 at T1-3, respectively) demonstrated differences in fecal metabolite profiles between subjects at all time points (Figure 2(a)). There were interindividual differences in the shifting pattern from T1 to T3 over time. The plots for each subject showed that the metabolite profile at T3 (2 weeks after cessation of ED) was still different from that at T1 (Figure 2(b)). PCA plots with the top3 loadings of PC1 (red arrows) and PC2 (blue arrows) showed that the impact of ED on the gut metabolites varies among subjects (Figure 2(c)).  Table 3 presents the 21 metabolites that showed significant differences between the time points, T1-3, among the 316 metabolites. The 21 metabolites demonstrated various changing patterns (Figure 3). Among them, 7 metabolites (butyric acid, 2,5-dihydroxybenzoic acid, asparagine, heptanoic acid, N-acetylglucosamine 1-phosphate, cadaverine, and isoglutamic acid) continued to decrease or increase even after the withdrawal of ED. The statistical results for all metabolites are shown in Table S3.

### 3.3. The Correlations between the Gut Bacteria and the Intestinal Metabolites

The correlations between the gut bacteria and the fecal metabolites were examined using all of the 12 samples at the OTU, genus, family, and phylum levels, respectively (Table S4). Furthermore, since the existence of samples that did not contain a specific microbe (e.g., the genus *Subdoligranulum* was not detected in 3 samples out of 12 samples) could influence the statistical correlations between the microbe and metabolites, the correlation analyses excluding the samples that did not contain each taxon were also performed (Table S5). The representative correlations between bacteria and metabolites at the OTU, genus, family, and phylum levels (Spearman correlation coefficient ≥ 0.9 or ≤-0.9, *p* < 0.01, and *q* < 0.01) are shown in Table S6. Several studies suggest that short-chain fatty acids (SCFAs) and bile acids (BAs) are involved in the pathogenesis of CD [27, 28]. Butyric acid and propionic acid are thought to have anti-inflammatory potentials. The correlations between these SCFAs and the gut bacterial OTUs were investigated.  Table 4 presents the significant positive and negative correlations (*p* < 0.05). The representative scatter plots for positive correlations (Spearman correlation coefficient ≥ 0.9) and for negative correlations (the top 5 strongest correlations with Spearman correlation coefficient ≤ −0.9) among these significant correlations are shown in Figure S2a. Significant correlations between butyric acid/propionic acid and gut bacteria at the genus, family, and phylum levels were also observed (Table S7). The metabolome analysis panel in the present study included cholic acid, deoxycholic acid, glycocholic acid, and taurocholic acid as primary and secondary BA. The correlations between these bile acids and the gut bacterial OTUs were examined. The significant positive and negative correlations in each bile acid (*p* < 0.05) are shown in Table 4. Figure S2b presents the representative scatter plot for the correlation with Spearman correlation coefficient ≥ 0.9 among these significant correlations. Significant correlations between the BAs and gut bacteria at the genus, family, and phylum levels were also observed (Table S8).

## 4. Discussion

The present study demonstrates that ED intervention (900 kcal/day) alters the gut bacterial compositions and certain intestinal metabolites, the patterns vary among subjects, and some effects persisted 2 weeks after the cessation of ED. Our study design analyzing the gut environment (bacterial compositions but also metabolites) in 4 healthy individuals over time provides important insights for a number of reasons. First, it is crucial to examine the human samples to better understand the human gut microbiota because there is a definite difference in the gut microbiome between human and animal models [29]. Second, since intestinal inflammation can influence the gut microbial compositions and ED can lessen the inflammation, we need to analyze healthy subjects excluding the influence of the inflammation as a confounding factor to directly assess the physiological impacts of ED on the human gut environment. Therefore, although it is recently reported that ED alters the gut bacterial structure in healthy mice [30], the study using healthy human samples is needed. Furthermore, one of the major advantages in the present study is investigating not only microbiome structure but also functions. We believe that tracking human subjects over time is another major advantage of this study. Given there are large interindividual variations in the gut microbial compositions among healthy adults [19, 31], a cross-sectional study of microbial membership could be useful but is not optimal for identifying disease-related microbes in the gut microbiota. Various factors, such as age [32, 33], sex [13, 34], diet [11–14], body mass index [13, 34], and genetic background [35, 36], can influence the gut microbiota, and there is a limitation in strictly controlling confounding factors in the human microbiome study. Therefore, in the present study, we employed a prospective longitudinal study design where subjects can serve as their own controls. Furthermore, since we tracked the subjects after the withdrawal of ED, we could observe the lasting impact of ED on the gut microbiota. Today, 16S rRNA gene amplicon sequencing analysis is widely used in the study of the gut microbiome, and this methodology is useful to investigate the bacterial composition. However, the functional information of the gut microbiota cannot be obtained with the 16S rRNA gene analysis, while the interactions between the gut microbes and the host immune system via metabolites are crucial for the host health [15, 16]. Hence, we performed the metabolomic analysis to investigate the impact of ED on the intestinal metabolites. We believe it is important to understand the gut microbiota as an environment from the viewpoint of not only the structure but also the function.

Our results showed large interindividual differences at all time points in the bacterial composition as well as the metabolite profile. In addition, the changes over time were not consistent among the subjects. These findings suggest that the alterations induced by ED may be affected by individuals' characteristics at the onset. If there still be some bacteria or metabolites that show the common shifting pattern across subjects, they may be key factors in the effect of ED on the gut environment. In this point of view, considering the previous reports on the involvement of SCFAs and BAs in the pathogenesis of CD [27, 28], it is interesting that butyric acid (*p* = 0.0183), deoxycholic acid (*p* = 0.0183), glycocholic acid (*p* = 0.0388), and taurocholic acid (*p* = 0.0388) were included in the metabolites showing significant differences between time points among 4 subjects. It also should be noted that the genera *[Ruminococcus]_gnavus_group* and *Parabacteroides* demonstrated significant differences between time points (*p* = 0.0183 and *p* = 0.0498, respectively). *[Ruminococcus]_gnavus_group* was strongly correlated with propionic acid (anti-inflammatory SCFA), cholic acid (primary BA), glycocholic acid (primary BA), and taurocholic acid (primary BA), while *Parabacteroides* was with propionic acid. The bacteria can compensate their functions with each other while the different bacterial strains even in the same species can exert different functions. Therefore, there still be a possibility that butyric acid and deoxycholic acid are physiologically important even without significant correlations with specific bacteria.

This study has several limitations. First, since 1 female and 3 male subjects participated, the sex difference could not be assessed. Second, the backgrounds of subjects were different in many characteristics (age, sex, BMI, and lifestyle) and we could not identify what factor(s) contribute the interindividual difference in our results. Third, since this is a human observational study, the mechanisms of how the bacteria and metabolites that we detected contribute to the clinical efficacy remain unclear. Only the 900 kcal/day of ED was used in this study. It would be interesting to examine the dose-dependency of the shifts of the gut environment to determine the optimal dose of ED. A study including a control group without ED intervention would be helpful to assess the possible endogenous overtime shifts of the gut microbiota and to clarify the impact of ED better. Also, a study with a longer observation period is also expected to investigate the lasting effect of ED that is suggested in this study. In addition, since ED is used for CD in the clinical settings and further studies with CD patients are needed, there could be differences in the impact of ED on the gut microbiota between healthy subjects and CD patients and even among CD patients with/without active GI inflammation. Several studies analyzing pediatric CD presented that EEN reduced the diversity of the gut microbiome [17]. However, Svolos et al. just recently reported that EEN but also their personalized diet for CD patients which is similar to EEN nutritionally did not change the diversity of the gut microbiome in healthy adult volunteers, while the personalized diet mimicking EEN showed the clinical efficacy for pediatric CD [37]. The microbial diversity did not change in our present study using 900 kcal/day of EN. This difference in the diversity shifts could be due to various differences in the analysis groups, e.g., CD vs. healthy and pediatric vs. adult subjects.

## 5. Conclusions

The present study demonstrated that 900 kcal/day of ED alters the gut bacterial composition but also the intestinal metabolites over time in healthy individuals. We observed that the shifts induced by ED vary among subjects, while some bacteria and metabolites are changed by ED across the subjects. The impact of ED on the gut environment is persistent at 2 weeks after the withdrawal. Further studies are needed to identify specific bacteria and metabolites and to understand the underlying mechanisms that are involved in the clinical efficacy of ED.

## Figures and Tables

**Table 1:** Background and nutrition of subjects.

Subject ID	K1	K2	K3	K4
Sex	Male	Male	Female	Male
Age	51	41	36	34
BMI	26.6	19.6	19.5	27.0
PMH	n/a	n/a	n/a	n/a
Nutrition				
T0^†^: total (kcal/day)	1827	1554	1314	2148
PFC ratio	13 : 27 : 60	16 : 31 : 53	13 : 35 : 52	15 : 39 : 46
T1^†^: total (kcal/day)	1480	1086	1711	1567
PFC ratio	18 : 38 : 44	15 : 29 : 56	13 : 34 : 53	16 : 30 : 54
T2^†^: total (kcal/day)	1581	1985	1846	1963
PFC ratio	18 : 22 : 60	15 : 19 : 66	16 : 16 : 68	16 : 17 : 67
T3^†^: total (kcal/day)	911	1446	1131	1998
PFC ratio	17 : 48 : 35	14 : 32 : 54	15 : 35 : 50	13 : 38 : 49

BMI: body mass index; PMH: past medical history; PFC ratio: protein : fat : carbohydrate ratio. ^†^The average of nutrition in 3 days before each time point based on subjects' diet record.

**Table 2:** Nutritional composition of the elemental diet (Elental®: 80 g, 300 kcal/package).

Composition	Amount
Nitrogen source	
Amino acids	14.1 g (17.6%)
Carbohydrate	
Dextrin	63.4 g (79.3%)
Fat	
Soy bean oil	0.51 g (0.6%)
Electrolytes	(2.0%)
Vitamins	14 types of vitamins

**Table 3:** The fecal metabolites exhibiting significant changes over time.

Metabolite	*p* value^†^
Butyric acid	0.0183
Deoxycholic acid	0.0183
FAD divalent	0.0183
Guanine	0.0183
2,5-Dihydroxybenzoic acid	0.0224
1-Methyl-4-imidazoleacetic acid	0.0224
Flavin mononucleotide	0.0381
Imidazole-4-acetic acid	0.0381
Asparagine	0.0381
Spermidine	0.0388
Proline	0.0388
N-Acetylglucosamine 1-phosphate	0.0388
Heptanoic acid	0.0388
8-Anilino-1-naphthalenesulfonic acid	0.0388
Glycocholic acid	0.0388
Taurocholic acid	0.0388
Carnitine	0.0388
Thymidine	0.0388
Isoglutamic acid	0.0498
Cadaverine	0.0498
S-Sulfocysteine	0.0498

^†^Friedman test.

**Table 4:** Significant correlations between bacterial OTUs and butyric acid, propionic acid, or bile acids.

OTU	Metabolite	Correlation coefficient	*p* value	*q* value	Sample size	Taxonomy
DQ797420.1.1394	Butyric acid	-0.899	0.015	0.215	6	*Alistipes* genus
CZBZ01000002.727652.729157	Butyric acid	-0.812	0.050	0.337	6	*Alistipes* genus
DQ793815.1.1392	Butyric acid	-0.812	0.050	0.337	6	*Alistipes* genus
FJ512477.1.1384	Butyric acid	-0.720	0.029	0.279	9	*Bacteroides* genus

HQ781065.1.1445	Propionic acid	0.975	4.8*E* − 03	0.146	5	*[Ruminococcus]_gnavus_group*
DQ806546.1.1397	Propionic acid	-1.000	2.8*E* − 03	0.128	6	*Alistipes* genus
CZBZ01000002.727652.729157	Propionic acid	-0.986	3.1*E* − 04	0.055	6	*Alistipes* genus
DQ793815.1.1392	Propionic acid	-0.986	3.1*E* − 04	0.055	6	*Alistipes* genus
DQ797420.1.1394	Propionic acid	-0.986	3.1*E* − 04	0.055	6	*Alistipes* genus
DQ798359.1.1394	Propionic acid	-0.986	3.1*E* − 04	0.055	6	*Alistipes* genus
DQ807419.1.1397	Propionic acid	-0.950	9.0*E* − 05	0.036	9	*Bacteroides* genus
KF842061.1.1404	Propionic acid	-0.946	1.2*E* − 04	0.039	9	*Bacteroides* genus
AJ518872.1.1414	Propionic acid	-0.943	1.7*E* − 02	0.215	6	*Alistipes* genus
FJ372122.1.1340	Propionic acid	-0.943	1.7*E* − 02	0.215	6	*Alistipes* genus
HQ760186.1.1442	Propionic acid	-0.941	1.5*E* − 04	0.046	9	*Bacteroides* genus
DQ794205.1.1401	Propionic acid	-0.937	1.9*E* − 04	0.050	9	*Bacteroides* genus
EU764029.1.1357	Propionic acid	-0.929	2.9*E* − 04	0.055	9	*Bacteroides* genus
HQ761547.1.1439	Propionic acid	-0.922	1.1*E* − 03	0.092	8	*Bacteroides* genus
DQ794515.1.1397	Propionic acid	-0.895	1.1*E* − 03	0.092	9	*Bacteroides* genus
DQ455907.1.1457	Propionic acid	-0.879	1.8*E* − 03	0.108	9	*Bacteroides* genus
EU763017.1.1357	Propionic acid	-0.879	1.8*E* − 03	0.108	9	*Bacteroides* genus
KF842604.1.1379	Propionic acid	-0.867	4.5*E* − 03	0.146	9	*Bacteroides* genus
DQ805835.1.1380	Propionic acid	-0.850	6.1*E* − 03	0.158	9	*Lachnospiraceae_UCG-004* genus
DQ456055.1.1452	Propionic acid	-0.833	8.3*E* − 03	0.172	9	*Bacteroides* genus
DQ824162.1.1399	Propionic acid	-0.765	1.6*E* − 02	0.215	9	*Bacteroides* genus
CDYJ01035375.5063.6592	Propionic acid	-0.717	3.7*E* − 02	0.303	9	*Ruminiclostridium_5* genus
CDZU01017197.3469.4972	Propionic acid	-0.686	4.1*E* − 02	0.317	9	*Odoribacter* genus

D86187.1.1513	Cholic acid	-0.833	0.015	0.215	8	*Bifidobacterium* genus
DQ824162.1.1399	Cholic acid	-0.681	0.044	0.331	9	*Bacteroides* genus

HQ781065.1.1445	Glycocholic acid	0.975	0.005	0.146	5	*[Ruminococcus]_gnavus_group*
DQ824162.1.1399	Glycocholic acid	-0.773	0.015	0.215	9	*Bacteroides* genus
HQ760186.1.1442	Glycocholic acid	-0.773	0.015	0.215	9	*Bacteroides* genus
KF842061.1.1404	Glycocholic acid	-0.770	0.015	0.215	9	*Bacteroides* genus
EU764029.1.1357	Glycocholic acid	-0.753	0.019	0.234	9	*Bacteroides* genus
DQ807419.1.1397	Glycocholic acid	-0.748	0.02	0.243	9	*Bacteroides* genus
DQ455907.1.1457	Glycocholic acid	-0.711	0.032	0.289	9	*Bacteroides* genus
DQ794205.1.1401	Glycocholic acid	-0.695	0.038	0.305	9	*Bacteroides* genus

EU768720.1.1347	Taurocholic acid	-0.762	0.037	0.303	8	*Bifidobacterium* genus
DQ824162.1.1399	Taurocholic acid	-0.740	0.023	0.257	9	*Bacteroides* genus
HQ760186.1.1442	Taurocholic acid	-0.731	0.025	0.267	9	*Bacteroides* genus
DQ805835.1.1380	Taurocholic acid	-0.717	0.037	0.303	9	*Lachnospiraceae_UCG-004 genus*
DQ455907.1.1457	Taurocholic acid	-0.711	0.032	0.289	9	*Bacteroides* genus
EU764029.1.1357	Taurocholic acid	-0.703	0.035	0.300	9	*Bacteroides* genus
KF842061.1.1404	Taurocholic acid	-0.669	0.049	0.337	9	*Bacteroides* genus

## Data Availability

The accession numbers for data in this paper are Submission: DRA008980, BioProject: PRJDB8759, BioSample: SAMD00184558-SAMD00184566, SAMD00185844-SAMD00185846,Experiment: DRX182461-DRX182472, Run: DRR192006-DRR192017.

## Supplementary Materials

Supplementary 1 Figure S1: study design to investigate the impact of elemental diet on the human gut microbiota.

Supplementary 2 Figure S2: the representative correlations between the gut bacterial operational taxonomical units and short-chain fatty acids/bile acids.

Supplementary 3 Table S1: taxonomical annotations and relative abundances of operational taxonomical units (OTUs) observed in the present study.

Supplementary 4 Table S2: the longitudinal shifts of the gut bacterial compositions (OTUs, genera, and a family with *p* < 1.00 (Friedman test)).

Supplementary 5 Table S3: statistics for longitudinal changes of the 316 fecal metabolites analyzed in the present study (Friedman test).

Supplementary 6 Table S4: spearman correlations between the gut bacteria and the intestinal metabolites (the OTU, genus, family, and phylum level in each tab).

Supplementary 7 Table S5: spearman correlations between the gut bacteria and the intestinal metabolites (the OTU, genus, family, and phylum level in each tab).

Supplementary 8 Table S6: representative correlations between bacteria and metabolites (Spearman correlation coefficient ≥ 0.9 or ≤-0.9, *p* < 0.01, and *q* < 0.01).

Supplementary 9 Table S7: significant correlations between bacterial taxonomy and butyric acid/propionic acid.

Supplementary 10 Table S8: significant correlations between bacterial taxonomy and bile acids.
