# Supplementary material for: Half-Elemental Diet Shifts the Human Intestinal Bacterial Compositions and Metabolites: A Pilot Study with Healthy Individuals
Source: Gastroenterol Res Pract. 2020 Aug 6;2020:7086939. doi: 10.1155/2020/7086939 (PMC7428940; doi:10.1155/2020/7086939)
Supplement: Supplementary 1 — Figure S1: study design to investigate the impact of elemental diet on the human gut microbiota. [file 7086939.f1.docx]

**Figure S1. Study design to investigate the impact of elemental diet on the human gut microbiota.**

Four healthy volunteers participated in the present study. After the registration (T0; week -2), the subjects continued their dietary habits for 2 weeks. At 2 weeks later (T1; week 0), the subject started 900 kcal/day of elemental diet (ED) and reduced dietary calorie intake by 900 kcal/day so that the daily calorie intake did not change in total. They stopped ED and resumed their original dietary habits at week 2 (T2). The observation was completed at week 4 (T3). Fecal samples and blood samples were collected from the subjects at the time points, T1-3.
