# Supplementary material for: Half-Elemental Diet Shifts the Human Intestinal Bacterial Compositions and Metabolites: A Pilot Study with Healthy Individuals
Source: Gastroenterol Res Pract. 2020 Aug 6;2020:7086939. doi: 10.1155/2020/7086939 (PMC7428940; doi:10.1155/2020/7086939)
Supplement: Supplementary 2 — Figure S2: the representative correlations between the gut bacterial operational taxonomical units and short-chain fatty acids/bile acids. [file 7086939.f2.docx]

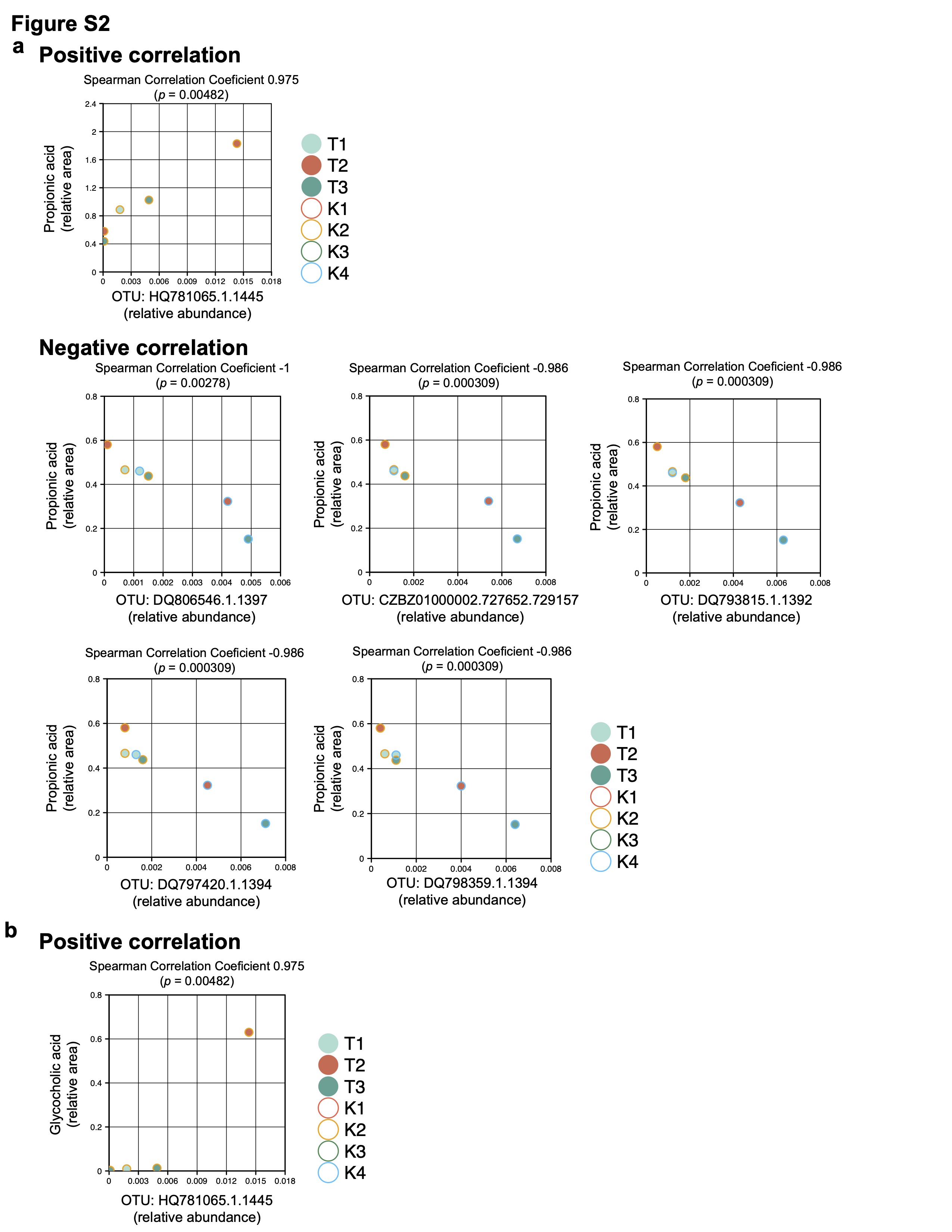


**Figure S2. The representative correlations between the gut bacterial operational taxonomical units and short-chain fatty acids/bile acids.**

(a) The representative scatter-plots for significant positive correlations (Spearman correlation coefficient ≥ 0.9) and significant negative correlations (the top 5 strongest correlations

with Spearman correlation coefficient ≤ - 0.9) between bacterial operational taxonomical units (OTUs) and short-chain fatty acids. (b) The representative scatter-plot (Spearman correlation coefficient ≥ 0.9) for the significant correlation between bacterial OTUs and bile acids.
