# Supplementary material for: Half-Elemental Diet Shifts the Human Intestinal Bacterial Compositions and Metabolites: A Pilot Study with Healthy Individuals
Source: Gastroenterol Res Pract. 2020 Aug 6;2020:7086939. doi: 10.1155/2020/7086939 (PMC7428940; doi:10.1155/2020/7086939)
Supplement: Supplementary 4 — Table S2: the longitudinal shifts of the gut bacterial compositions (OTUs, genera, and a family with p < 1.00 (Friedman test)). [file 7086939.f4.docx]

Table S2. The longitudinal shifts of the gut bacterial compositions [OTUs, genera, and a family with *p* < 1.00 (Friedman test)]

| **Bacterial OTU** | ***p* value** |
| --- | --- |
| D86187.1.1513 | 0.0970 |
| EU768720.1.1347 | 0.0970 |
| CVOZ01000063.228971.230480 | 0.0970 |
| FJ503329.1.1367 | 0.1353 |
| HQ793781.1.1478 | 0.1561 |
| HQ781065.1.1445 | 0.1561 |
| DQ795359.1.1391 | 0.1561 |
| DQ797420.1.1394 | 0.1561 |
| DQ798502.1.1389 | 0.1561 |
| DQ808021.1.1420 | 0.1561 |
| EF071375.1.1507 | 0.1561 |
| FJ509235.1.1351 | 0.1561 |
| FJ368451.1.1358 | 0.1561 |
| FJ365729.1.1352 | 0.1561 |
| HQ793825.1.1481 | 0.1561 |
| FJ676367.1.1369 | 0.2231 |
| DQ806546.1.1397 | 0.2231 |
| FJ372122.1.1340 | 0.2231 |
| FJ369674.1.1367 | 0.2231 |
| DQ793815.1.1392 | 0.2231 |
| FJ365354.1.1352 | 0.2231 |
| LT223639.1.1437 | 0.2231 |
| DQ798359.1.1394 | 0.2231 |
| CZBZ01000002.727652.729157 | 0.2231 |
| AJ518872.1.1414 | 0.2231 |
| DQ057383.1.1395 | 0.2636 |
| DQ824162.1.1399 | 0.3067 |
| EU768719.1.1355 | 0.3067 |
| HQ760186.1.1442 | 0.3067 |
| HQ795363.1.1466 | 0.3679 |
| FM873430.1.1507 | 0.3679 |
| JQ940611.1.1450 | 0.3679 |
| FJ512477.1.1384 | 0.3679 |
| FJ512224.1.1379 | 0.3679 |
| KF071484.1.1353 | 0.3679 |
| FJ508627.1.1357 | 0.3679 |
| KF072758.1.1379 | 0.3679 |
| FJ440034.1.1501 | 0.3679 |
| FJ440083.1.1451 | 0.3679 |
| KF842061.1.1404 | 0.3679 |
| FJ370984.1.1398 | 0.3679 |
| KF842100.1.1418 | 0.3679 |
| KF842215.1.1420 | 0.3679 |
| KF842513.1.1417 | 0.3679 |
| FJ364668.1.1255 | 0.3679 |
| KF842600.1.1379 | 0.3679 |
| FJ364053.1.1357 | 0.3679 |
| FJ503238.1.1390 | 0.3679 |
| GQ491553.1.1336 | 0.3679 |
| JQ186816.1.1359 | 0.3679 |
| GQ493584.1.1333 | 0.3679 |
| HQ785949.1.1477 | 0.3679 |
| HQ785621.1.1430 | 0.3679 |
| HQ796231.1.1425 | 0.3679 |
| HQ782057.1.1433 | 0.3679 |
| HQ796241.1.1433 | 0.3679 |
| HQ796332.1.1437 | 0.3679 |
| HQ776651.1.1449 | 0.3679 |
| HQ775107.1.1452 | 0.3679 |
| HQ804231.1.1422 | 0.3679 |
| GQ492248.1.1368 | 0.3679 |
| HQ761547.1.1439 | 0.3679 |
| FJ363260.1.1341 | 0.3679 |
| HQ810977.1.1427 | 0.3679 |
| HQ754391.1.1413 | 0.3679 |
| JF057596.1.1380 | 0.3679 |
| GQ898364.1.1484 | 0.3679 |
| JQ184675.1.1362 | 0.3679 |
| GQ897903.1.1487 | 0.3679 |
| GQ897155.1.1487 | 0.3679 |
| GQ493883.1.1371 | 0.3679 |
| HQ760236.1.1426 | 0.3679 |
| FJ362947.1.1366 | 0.3679 |
| AB510700.1.1488 | 0.3679 |
| EU778973.1.1417 | 0.3679 |
| EU779071.1.1414 | 0.3679 |
| DQ797812.1.1399 | 0.3679 |
| DQ797047.1.1397 | 0.3679 |
| DQ795626.1.1389 | 0.3679 |
| DQ793241.1.1399 | 0.3679 |
| DQ456076.1.1438 | 0.3679 |
| CZCA01000489.3432.4942 | 0.3679 |
| CEAH01024297.182.1686 | 0.3679 |
| CDYT01008935.2544.4050 | 0.3679 |
| CDYK01001058.46302.47796 | 0.3679 |
| AY986194.1.1358 | 0.3679 |
| AY986193.1.1358 | 0.3679 |
| AY986162.1.1358 | 0.3679 |
| AY986067.1.1358 | 0.3679 |
| AY984258.1.1335 | 0.3679 |
| AY977418.1.1358 | 0.3679 |
| AY977071.1.1335 | 0.3679 |
| AY975423.1.1358 | 0.3679 |
| AJ867037.1.1462 | 0.3679 |
| ADMB01000074.90.1632 | 0.3679 |
| ABVO01000045.3575.5089 | 0.3679 |
| DQ823742.1.1413 | 0.3679 |
| DQ823980.1.1393 | 0.3679 |
| EU767901.1.1354 | 0.3679 |
| EF401572.1.1475 | 0.3679 |
| EF404740.1.1497 | 0.3679 |
| EF404342.1.1497 | 0.3679 |
| EF403836.1.1489 | 0.3679 |
| EU531977.1.1448 | 0.3679 |
| EU531995.1.1462 | 0.3679 |
| EF403635.1.1517 | 0.3679 |
| EF403475.1.1493 | 0.3679 |
| EU009791.1.1480 | 0.3679 |
| EU136695.1.1991 | 0.3679 |
| EF403397.1.1491 | 0.3679 |
| EF403317.1.1491 | 0.3679 |
| EF403163.1.1517 | 0.3679 |
| EF401870.1.1477 | 0.3679 |
| EU767561.1.1352 | 0.3679 |
| EU767611.1.1349 | 0.3679 |
| LFQU01000071.20.1554 | 0.3679 |
| EU778953.1.1414 | 0.3679 |
| EU764029.1.1357 | 0.3679 |
| EU763017.1.1357 | 0.5292 |
| EU531898.1.1460 | 0.6065 |
| DQ798024.1.1383 | 0.6065 |
| CDYJ01035375.5063.6592 | 0.7165 |
| DQ456055.1.1452 | 0.7165 |
| DQ805835.1.1380 | 0.7165 |
| GU361827.1.1480 | 0.7165 |
| KF842604.1.1379 | 0.7165 |
| CDZU01017197.3469.4972 | 0.7165 |
| DQ455907.1.1457 | 0.7613 |
| DQ794515.1.1397 | 0.7613 |
| DQ798958.1.1391 | 0.7613 |
| FJ370779.1.1389 | 0.8669 |
| DQ807419.1.1397 | 0.9048 |
| **Genus** |  |
| [Ruminococcus]_gnavus_group | 0.0183 |
| Parabacteroides | 0.0498 |
| Tyzzerella | 0.0970 |
| [Eubacterium]_coprostanoligenes_group | 0.0970 |
| Lachnospiraceae_ND3007_group | 0.0970 |
| Ruminiclostridium_9 | 0.0970 |
| Lachnospira | 0.1266 |
| Faecalitalea | 0.1353 |
| Bilophila | 0.1462 |
| Barnesiella | 0.1482 |
| Anaerotruncus | 0.1482 |
| Ruminococcaceae_UCG-013 | 0.1653 |
| Roseburia | 0.1738 |
| Faecalibacterium | 0.1738 |
| Christensenellaceae_R-7_group | 0.1778 |
| Sutterella | 0.1889 |
| Erysipelotrichaceae_UCG-003 | 0.2231 |
| Tyzzerella_4 | 0.2466 |
| [Eubacterium]_rectale_group | 0.2466 |
| Ruminococcaceae_NK4A214_group | 0.2725 |
| [Eubacterium]_ventriosum_group | 0.3067 |
| Fusobacterium | 0.3067 |
| Subdoligranulum | 0.3067 |
| Phascolarctobacterium | 0.3679 |
| Prevotellaceae_NK3B31_group | 0.3679 |
| Megamonas | 0.3679 |
| Acidaminococcus | 0.3679 |
| Holdemanella | 0.3679 |
| Ruminococcus_2 | 0.3679 |
| unassigned Clostridiales_vadinBB60_group | 0.3679 |
| Anaerostipes | 0.3679 |
| Bacteroides | 0.3679 |
| Blautia | 0.3679 |
| uncultured Lachnospiraceae | 0.4204 |
| Alistipes | 0.4204 |
| Ruminococcaceae_UCG-002 | 0.4204 |
| Bifidobacterium | 0.4204 |
| Ruminococcus_1 | 0.4724 |
| Lachnoclostridium | 0.4724 |
| [Ruminococcus]_gauvreauii_group | 0.4724 |
| Streptococcus | 0.4724 |
| Tyzzerella_3 | 0.5258 |
| [Eubacterium]_hallii_group | 0.5488 |
| Escherichia-Shigella | 0.6065 |
| Collinsella | 0.6271 |
| Dorea | 0.6271 |
| uncultured Ruminococcaceae | 0.7165 |
| Lachnospiraceae_NK4A136_group | 0.7165 |
| Fusicatenibacter | 0.7515 |
| Shuttleworthia | 0.7613 |
| Intestinibacter | 0.7788 |
| Butyricicoccus | 0.7788 |
| [Ruminococcus]_torques_group | 0.7788 |
| Flavonifractor | 0.7788 |
| Odoribacter | 0.7788 |
| uncultured Porphyromonadaceae | 0.7788 |
| Ruminiclostridium_5 | 0.7788 |
| Lachnospiraceae_UCG-004 | 0.7788 |
| Prevotella_9 | 0.8071 |
| Parasutterella | 0.9048 |
| Erysipelatoclostridium | 0.9311 |
| Eggerthella | 0.9355 |
| Sellimonas | 0.9355 |
| **Family** |  |
| Christensenellaceae | 0.097 |
| Erysipelotrichaceae | 0.105 |
| Alcaligenaceae | 0.105 |
| Ruminococcaceae | 0.174 |
| Desulfovibrionaceae | 0.223 |
| Enterobacteriaceae | 0.282 |
| Prevotellaceae | 0.282 |
| Fusobacteriaceae | 0.307 |
| Bacteroidaceae | 0.368 |
| Clostridiales_vadinBB60_group | 0.368 |
| Lachnospiraceae | 0.368 |
| Porphyromonadaceae | 0.368 |
| Bifidobacteriaceae | 0.420 |
| Rikenellaceae | 0.420 |
| Coriobacteriaceae | 0.472 |
| Streptococcaceae | 0.472 |
| Veillonellaceae | 0.607 |
| Acidaminococcaceae | 0.717 |
| Peptostreptococcaceae | 0.779 |
| Family_XIII | 0.936 |
| **Phylum** |  |
| Fusobacteria | 0.3067 |
| Proteobacteria | 0.3679 |
| Actinobacteria | 0.4204 |
| Bacteroidetes | 0.7788 |
| Firmicutes | 0.7788 |
