# Supplementary material for: Half-Elemental Diet Shifts the Human Intestinal Bacterial Compositions and Metabolites: A Pilot Study with Healthy Individuals
Source: Gastroenterol Res Pract. 2020 Aug 6;2020:7086939. doi: 10.1155/2020/7086939 (PMC7428940; doi:10.1155/2020/7086939)
Supplement: Supplementary 5 — Table S3: statistics for longitudinal changes of the 316 fecal metabolites analyzed in the present study (Friedman test). [file 7086939.f5.docx]

Table S3. Statistics for longitudinal changes of the 316 fecal metabolites analyzed in the present study (Friedman test)

| **Metabolites** | ***p* value** |
| --- | --- |
| Butyric acid | 0.0183 |
| Deoxycholic acid | 0.0183 |
| FAD_divalent | 0.0183 |
| Guanine | 0.0183 |
| 2,5-Dihydroxybenzoic acid | 0.0224 |
| 1-Methyl-4-imidazoleacetic acid | 0.0224 |
| FMN | 0.0381 |
| Imidazole-4-acetic acid | 0.0381 |
| Asn | 0.0381 |
| Spermidine | 0.0388 |
| Pro | 0.0388 |
| N-Acetylglucosamine 1-phosphate | 0.0388 |
| Heptanoic acid | 0.0388 |
| 8-Anilino-1-naphthalenesulfonic acid | 0.0388 |
| Glycocholic acid | 0.0388 |
| Taurocholic acid | 0.0388 |
| Carnitine | 0.0388 |
| Thymidine | 0.0388 |
| Isoglutamic acid | 0.0498 |
| Cadaverine | 0.0498 |
| S-Sulfocysteine | 0.0498 |
| Ribulose 5-phosphate | 0.0597 |
| Cys | 0.0597 |
| Glucose 1-phosphate | 0.0597 |
| threo-β-Methylaspartic acid | 0.0597 |
| Serotonin | 0.0608 |
| Tyrosine methyl ester | 0.0608 |
| Acetyl CoA_divalent | 0.0764 |
| Undecanoic acid | 0.0764 |
| Isobutylamine | 0.0849 |
| 3-Hydroxybutyric acid | 0.0859 |
| Pyridoxamine 5'-phosphate | 0.0859 |
| Piperidine | 0.0970 |
| Fructose 6-phosphate | 0.0970 |
| Shikimic acid | 0.0970 |
| Quinolinic acid | 0.1054 |
| 6-Phosphogluconic acid | 0.1054 |
| Propionic acid | 0.1054 |
| p-Hydroxybenzoic acid | 0.1054 |
| 2-Hydroxyvaleric acid | 0.1054 |
| Stachydrine | 0.1054 |
| Leu | 0.1054 |
| Methionine sulfoxide | 0.1054 |
| Histamine | 0.1054 |
| Glycerol 3-phosphate | 0.1054 |
| Glutaric acid | 0.1054 |
| Timolol | 0.1092 |
| Pyridoxine | 0.1092 |
| GABA | 0.1266 |
| Sulfotyrosine | 0.1266 |
| Carnosine | 0.1353 |
| Sinapic acid | 0.1353 |
| Diethylaminomalonic acid | 0.1353 |
| Isobutyryl CoA_divalent | 0.1353 |
| 10-Hydroxydecanoic acid | 0.1462 |
| trans-Zeatin | 0.1482 |
| Trimethylamine | 0.1482 |
| 6,8-Thioctic acid | 0.1561 |
| 2-Deoxyglucose 6-phosphate | 0.1561 |
| 5-Amino-4-oxovaleric acid | 0.1561 |
| Ethylacetimidate | 0.1561 |
| 2-Hydroxybutyric acid | 0.1561 |
| Oxamic acid | 0.1561 |
| Phthalic acid | 0.1653 |
| N-Methylglutamic acid | 0.1653 |
| Tyramine | 0.1738 |
| 5-Aminovaleric acid | 0.1738 |
| CMP | 0.1738 |
| Gly | 0.1738 |
| N-Acetylglucosamine 6-phosphate | 0.1738 |
| Glu | 0.1738 |
| Pelargonic acid | 0.1738 |
| Dodecanedioic acid | 0.1738 |
| Threonic acid | 0.1738 |
| Cyclohexylamine | 0.1738 |
| Glycerol | 0.1738 |
| Hexanoic acid | 0.1738 |
| Asp | 0.1738 |
| Creatine | 0.1738 |
| N-Acetylglutamine | 0.1738 |
| Taurine | 0.1738 |
| 4-Aminoindole | 0.1738 |
| 3-Phenylpropionic acid | 0.1738 |
| Saccharopine | 0.1738 |
| N-Acetylglutamic acid | 0.1738 |
| Citramalic acid | 0.1778 |
| 2-Quinolinecarboxylic acid | 0.2019 |
| Myristoleic acid | 0.2019 |
| UDP-glucuronic acid | 0.2231 |
| 8-Hydroxyoctanoic acid | 0.2231 |
| Formylanthranilic acid | 0.2231 |
| O-Acetylcarnitine | 0.2231 |
| Alloisoleucine | 0.2231 |
| 1-Methylhistamine | 0.2231 |
| Mucic acid | 0.2231 |
| NADH | 0.2231 |
| Metronidazole | 0.2574 |
| Sedoheptulose 7-phosphate | 0.2574 |
| N-Acetylputrescine | 0.2636 |
| Gluconic acid | 0.2636 |
| Cytosine | 0.2725 |
| N2-Acetylaminoadipic acid | 0.3067 |
| Imidazolelactic acid | 0.3067 |
| NADP+ | 0.3067 |
| Scopolamine | 0.3067 |
| Tryptamine | 0.3189 |
| 3-Methylguanine | 0.3679 |
| m-Hydroxybenzoic acid | 0.3679 |
| S-Methylmethionine | 0.3679 |
| Hordenine | 0.3679 |
| 3-Acetylacrylic acid | 0.3679 |
| 5-Hydroxylysine | 0.3679 |
| N-Acetylglycine | 0.3679 |
| Pterin | 0.3679 |
| Succinic acid | 0.3679 |
| Azetidine 2-carboxylic acid | 0.3679 |
| 6-Aminohexanoic acid | 0.3679 |
| Pipecolic acid | 0.3679 |
| 5-Methylcytosine | 0.3679 |
| Citraconic acid | 0.3679 |
| 2-Phenylethylamine | 0.3679 |
| 3-Ureidopropionic acid | 0.3679 |
| Anserine_divalent | 0.3679 |
| Homoserine | 0.3679 |
| 2,4-Diaminobutyric acid | 0.3679 |
| 3-Hydroxyanthranilic acid | 0.3679 |
| Val | 0.3679 |
| 5-Oxo-2-tetrahydrofurancarboxylic acid | 0.3679 |
| Hexamine | 0.3679 |
| 5-Oxoproline | 0.3679 |
| γ-Butyrobetaine | 0.3679 |
| N-Methylaspartic acid | 0.3679 |
| 2-Amino-2-methyl-1,3-propanediol | 0.3679 |
| Isethionic acid | 0.3679 |
| Thiamine diphosphate | 0.3679 |
| N-Methylalanine | 0.3679 |
| Urocanic acid | 0.3679 |
| Trehalose 6-phosphate | 0.3679 |
| S-Adenosylmethionine | 0.3679 |
| Butyrylcarnitine | 0.3679 |
| 3-Indoxylsulfuric acid | 0.3679 |
| Phosphoenolpyruvic acid | 0.3679 |
| γ-Glu-2-aminobutyric acid | 0.3679 |
| Xanthurenic acid | 0.3679 |
| 7,8-Dihydrobiopterin | 0.3679 |
| 3-Hydroxypropionic acid | 0.3679 |
| Uridine | 0.3679 |
| Syringic acid | 0.3679 |
| o-Hydroxyhippuric acid | 0.3679 |
| Ferulic acid | 0.3679 |
| Tetrahydrouridine | 0.3679 |
| β-Ala-Lys | 0.3679 |
| Quinic acid | 0.3679 |
| Citric acid | 0.3679 |
| Cysteic acid | 0.3679 |
| Azelaic acid | 0.3679 |
| 3-Phosphoglyceric acid | 0.3679 |
| Chelidonic acid | 0.3679 |
| Octanoylcarnitine | 0.3679 |
| Hippuric acid | 0.3679 |
| Argininosuccinic acid | 0.3679 |
| 5'-Deoxy-5'-methylthioadenosine | 0.3679 |
| Phenoxybenzamine | 0.3679 |
| Crotonic acid | 0.3679 |
| Isocitric acid | 0.3679 |
| Nalidixic acid | 0.3679 |
| CoA_divalent | 0.3679 |
| 3-Methoxytyrosine | 0.3679 |
| Citrulline | 0.3679 |
| S-Carboxymethylcysteine | 0.3679 |
| β-Tyr | 0.3679 |
| Mevalonic acid | 0.3679 |
| cis-4-Hydroxyproline | 0.3679 |
| Digalacturonic acid | 0.3679 |
| Adrenaline | 0.3679 |
| N,N-Dimethylhistidine | 0.3679 |
| cGMP | 0.3679 |
| Nω-Methylarginine | 0.3679 |
| N-Methylserotonin | 0.3679 |
| 2-(Creatinine-3-yl)propionic acid | 0.3679 |
| Glucosaminic acid | 0.3679 |
| Xanthosine | 0.3679 |
| p-Hydroxyphenylacetic acid | 0.3679 |
| 3-Hydroxy-3-methylglutaric acid | 0.3679 |
| N6-Acetyllysine | 0.4204 |
| Hydroxyproline | 0.4204 |
| Methoxamine | 0.4204 |
| Met | 0.4204 |
| Propionyl CoA_divalent | 0.4244 |
| Methyl sulfate | 0.4412 |
| 3,4-Dihydroxyphenylglycol | 0.4412 |
| N6,N6,N6-Trimethyllysine | 0.4724 |
| N1,N8-Diacetylspermidine | 0.4724 |
| Ile | 0.4724 |
| 4-(β-Acetylaminoethyl)imidazole | 0.4724 |
| Guanosine | 0.4724 |
| Tyr | 0.4724 |
| N-Acetylhistidine | 0.4724 |
| Adenosine | 0.4724 |
| Dyphylline | 0.4724 |
| N6-Methyllysine | 0.4724 |
| Carboxymethyllysine | 0.4724 |
| Arg | 0.4724 |
| Decanoic acid | 0.4724 |
| N-Acetylalanine | 0.4724 |
| β-Ala | 0.4724 |
| Octanoic acid | 0.4724 |
| Adipic acid | 0.4724 |
| 2-Hydroxyglutaric acid | 0.4724 |
| Cholic acid | 0.4724 |
| AMP | 0.4724 |
| Pimelic acid | 0.4724 |
| N2-Phenylacetylglutamine | 0.4724 |
| Malic acid | 0.4724 |
| Pantothenic acid | 0.4724 |
| Lauric acid | 0.4724 |
| Phenaceturic acid | 0.4724 |
| Nicotinic acid | 0.4724 |
| 4-Pyridoxic acid | 0.4724 |
| N-Acetylaspartic acid | 0.4724 |
| Suberic acid | 0.4724 |
| p-Hydroxymandelic acid | 0.4724 |
| Kynurenic acid | 0.4966 |
| Benzoic acid | 0.4966 |
| Homocarnosine | 0.5258 |
| Sarcosine | 0.5258 |
| 3-Guanidinopropionic acid | 0.5292 |
| N5-Ethylglutamine | 0.5292 |
| Formiminoglutamic acid | 0.5292 |
| N1-Acetylspermine | 0.6065 |
| trans-Cinnamic acid | 0.6065 |
| 2-Amino-2-(hydroxymethyl)-1,3-propanediol | 0.6065 |
| O-Acetylserine | 0.6065 |
| Uracil | 0.6065 |
| Hydroxyindole | 0.6065 |
| N-Carbamoylaspartic acid | 0.6271 |
| 2,6-Diaminopimelic acid | 0.6271 |
| dAMP | 0.6703 |
| 3-Aminopropane-1,2-diol | 0.7165 |
| Mannosamine | 0.7165 |
| 3-Amino-2-piperidone | 0.7165 |
| 6-Hydroxynicotinic acid | 0.7165 |
| 2-Isopropylmalic acid | 0.7613 |
| Homocysteic acid | 0.7613 |
| Ascorbate 2-sulfate | 0.7613 |
| Glyceric acid | 0.7613 |
| Trp | 0.7788 |
| N-Acetylmuramic acid | 0.7788 |
| Thr | 0.7788 |
| Homocitrulline | 0.7788 |
| UMP | 0.7788 |
| SDMA | 0.7788 |
| Terephthalic acid | 0.7788 |
| 5-Methoxyindoleacetic acid | 0.7788 |
| Ethyl glucuronide | 0.7788 |
| Hypoxanthine | 0.7788 |
| Adenine | 0.7788 |
| Ornithine | 0.7788 |
| Vanillylmandelic acid | 0.7788 |
| 5-Aminoindole | 0.7788 |
| 5-Hydroxyindoleacetic acid | 0.7788 |
| Thiamine | 0.7788 |
| Nicotinamide | 0.7788 |
| Inosine | 0.7788 |
| Homovanillic acid | 0.7788 |
| 5-Oxohexanoic acid | 0.7788 |
| Agmatine | 0.7788 |
| 3-(4-Hydroxyphenyl)propionic acid | 0.7788 |
| N8-Acetylspermidine | 0.7788 |
| Choline | 0.7788 |
| dTMP | 0.7788 |
| Putrescine | 0.7788 |
| Lys | 0.7788 |
| Phe | 0.7788 |
| 4-Acetamidobutanoic acid | 0.7788 |
| Ala | 0.7788 |
| His | 0.7788 |
| Cyclohexanecarboxylic acid | 0.7788 |
| 6-Hydroxyhexanoic acid | 0.7788 |
| Diethanolamine | 0.7788 |
| Ser | 0.7788 |
| Diphenylcarbazide | 0.8071 |
| Creatinine | 0.8187 |
| Ethanolamine | 0.8187 |
| ADP | 0.8187 |
| N-Acetylneuraminic acid | 0.8187 |
| Xanthine | 0.8669 |
| Normetanephrine | 0.8669 |
| 2-Hydroxy-4-methylvaleric acid | 0.8669 |
| Tetrahydropalmatine | 0.8669 |
| dTDP | 0.8669 |
| N-Acetylornithine | 0.8669 |
| Glucose 6-phosphate | 0.8669 |
| 1-Methylnicotinamide | 0.8669 |
| 3-Aminobutyric acid | 0.9048 |
| Glycerophosphocholine | 0.9048 |
| NMN | 0.9131 |
| Betonicine | 0.9260 |
| N-Acetyl-β-alanine | 0.9260 |
| N-Methylproline | 0.9355 |
| Prostaglandin E2 | 1.0000 |
| Gln | 1.0000 |
| 2-Methylserine | 1.0000 |
| NAD+ | 1.0000 |
| ADP-ribose | 1.0000 |
| Pyridoxamine | 1.0000 |
| Lactic acid | 1.0000 |
| 2-Hydroxyisobutyric acid | 1.0000 |
| Biotin | 1.0000 |
| Guanidinosuccinic acid | 1.0000 |
| UDP | 1.0000 |
| GMP | 1.0000 |
| Prostaglandin F2α | 1.0000 |
| N-Acetyllysine | 1.0000 |
| Isopropanolamine | 1.0000 |
| Sebacic acid | 1.0000 |
