# Supplementary material for: Half-Elemental Diet Shifts the Human Intestinal Bacterial Compositions and Metabolites: A Pilot Study with Healthy Individuals
Source: Gastroenterol Res Pract. 2020 Aug 6;2020:7086939. doi: 10.1155/2020/7086939 (PMC7428940; doi:10.1155/2020/7086939)
Supplement: Supplementary 8 — Table S6: representative correlations between bacteria and metabolites (Spearman correlation coefficient ≥ 0.9 or ≤-0.9, p < 0.01, and q < 0.01). [file 7086939.f8.docx]

Table S6. Representative correlations between bacteria and metabolites (Spearman correlation coefficient ≥ 0.9 or ≤ -0.9, *p* < 0.01, and *q* < 0.01)

| **OTU** | **Metabolite** | **Correlation**  **coefficient** | ***p* value** | ***q* value** | **Sample size** | **Taxonomy** |
| --- | --- | --- | --- | --- | --- | --- |
| FJ509235.1.1351 | γ-Glu-2-aminobutyric acid | 1 | 0 | 0 | 5 | *Faecalibacterium* genus |
| HQ781065.1.1445 | 2-Hydroxybutyric acid | 1 | 0 | 0 | 5 | [Ruminococcus]_gnavus_group |
| HQ781065.1.1445 | 3-Aminopropane-1,2-diol | 1 | 0 | 0 | 5 | [Ruminococcus]_gnavus_group |
| HQ781065.1.1445 | O-Acetylcarnitine | 1 | 0 | 0 | 5 | [Ruminococcus]_gnavus_group |
| DQ905920.1.1559 | N-Carbamoylaspartic acid | -1 | 0 | 0 | 4 | *Barnesiella* genus |
| DQ905920.1.1559 | Kynurenic acid | -1 | 0 | 0 | 4 | *Barnesiella* genus |
| DQ905920.1.1559 | Sulfotyrosine | -1 | 0 | 0 | 4 | *Barnesiella* genus |
| DQ905920.1.1559 | Met | -1 | 0 | 0 | 4 | *Barnesiella* genus |
| EU764029.1.1357 | Inosine | -0.99582 | 1.54E-08 | 1.63E-05 | 9 | *Bacteroides* genus |
| HQ760186.1.1442 | Inosine | -0.99163 | 1.75E-07 | 0.000154 | 9 | *Bacteroides* genus |
| HQ761547.1.1439 | Inosine | -0.99403 | 5.3E-07 | 0.000399 | 8 | *Bacteroides* genus |
| DQ455907.1.1457 | Inosine | -0.97909 | 4.27E-06 | 0.001965 | 9 | *Bacteroides* genus |
| EU763017.1.1357 | Ser | -0.97909 | 4.27E-06 | 0.001965 | 9 | *Bacteroides* genus |
| KF842061.1.1404 | Ser | -0.97909 | 4.27E-06 | 0.001965 | 9 | *Bacteroides* genus |
| KF842061.1.1404 | Inosine | -0.97909 | 4.27E-06 | 0.001965 | 9 | *Bacteroides* genus |
| EU763017.1.1357 | Citramalic acid | -0.9788 | 4.47E-06 | 0.001965 | 9 | *Bacteroides* genus |
| DQ807419.1.1397 | Ser | -0.97482 | 8.13E-06 | 0.003063 | 9 | *Bacteroides* genus |
| HQ760186.1.1442 | Ser | -0.97482 | 8.13E-06 | 0.003063 | 9 | *Bacteroides* genus |
| HQ760186.1.1442 | 2-Hydroxyvaleric acid | -0.96642 | 2.21E-05 | 0.007271 | 9 | *Bacteroides* genus |
| CDYJ01035375.5063.6592 | Mannosamine | -0.96624 | 2.25E-05 | 0.007271 | 9 | *Ruminiclostridium_5* genus |
| DQ807419.1.1397 | 6-Hydroxynicotinic acid | -0.96585 | 2.34E-05 | 0.007271 | 9 | *Bacteroides* genus |
| DQ455907.1.1457 | 2-Hydroxyvaleric acid | -0.96235 | 3.29E-05 | 0.00825 | 9 | *Bacteroides* genus |
| DQ794515.1.1397 | Ser | -0.96235 | 3.29E-05 | 0.00825 | 9 | *Bacteroides* genus |
| DQ794515.1.1397 | Inosine | -0.96235 | 3.29E-05 | 0.00825 | 9 | *Bacteroides* genus |
| EU764029.1.1357 | Ser | -0.96235 | 3.29E-05 | 0.00825 | 9 | *Bacteroides* genus |
| **Genus** |  |  |  |  |  |  |
| Bacteroides | 5-Aminovaleric acid | 0.923077 | 0 | 0 | 12 |  |
| Bacteroides | Creatine | 0.902098 | 0 | 0 | 12 |  |
| Parabacteroides | Spermidine | 0.909091 | 0 | 0 | 12 |  |
| Ruminococcus_1 | N-Acetylglutamine | 0.918182 | 0 | 0 | 11 |  |
| Ruminococcus_1 | N-Methylproline | 0.945455 | 0 | 0 | 11 |  |
| Prevotella_9 | Cholic acid | 0.97711 | 4.76E-08 | 4.06E-05 | 12 |  |
| Dorea | Guanosine | 0.931253 | 1.08E-05 | 0.007876 | 12 |  |
| Bifidobacterium | 5-Oxoproline | -0.93939 | 0 | 0 | 10 |  |
| Bifidobacterium | Trp | -0.96364 | 0 | 0 | 10 |  |
| Collinsella | 3-(4-Hydroxyphenyl) propionic acid | -0.95152 | 0 | 0 | 10 |  |
| Collinsella | Ser | -0.97576 | 0 | 0 | 10 |  |
| Roseburia | Cholic acid | -0.90909 | 0 | 0 | 12 |  |
| Ruminococcus_1 | Cholic acid | -0.92727 | 0 | 0 | 11 |  |
| Ruminococcus_1 | Stachydrine | -0.93636 | 0 | 0 | 11 |  |
| Ruminococcus_1 | 5'-Deoxy-5'-methylthioadenosine | -0.95455 | 0 | 0 | 11 |  |
| Alistipes | Homovanillic acid | -0.95622 | 1.18E-06 | 0.000778 | 12 |  |
| Prevotella_9 | N-Methylproline | -0.94349 | 4.13E-06 | 0.002456 | 12 |  |
| Alistipes | Phe | -0.94221 | 4.61E-06 | 0.002492 | 12 |  |
| Ruminococcaceae_UCG-002 | Putrescine | -0.95417 | 5.17E-06 | 0.002566 | 11 |  |
| Shuttleworthia | N8-Acetylspermidine | -0.97482 | 8.13E-06 | 0.003723 | 9 |  |
| Alistipes | 3-Hydroxybutyric acid | -0.92614 | 1.53E-05 | 0.006494 | 12 |  |
| Alistipes | 2-Hydroxyvaleric acid | -0.92119 | 2.09E-05 | 0.008223 | 12 |  |
| Shuttleworthia | 3-(4-Hydroxyphenyl) propionic acid | -0.96642 | 2.21E-05 | 0.008223 | 9 |  |
| Ruminococcus_1 | 3-Hydroxybutyric acid | -0.93439 | 2.53E-05 | 0.008606 | 11 |  |
| Ruminococcus_1 | Met | -0.93394 | 2.6E-05 | 0.008606 | 11 |  |
| Alistipes | 2-Methylserine | -0.91634 | 2.8E-05 | 0.008774 | 12 |  |
| Alistipes | Putrescine | -0.91419 | 3.17E-05 | 0.009428 | 12 |  |
| Bifidobacterium | 5-Oxoproline | -0.93939 | 0 | 0 | 10 |  |
| Bifidobacterium | Trp | -0.96364 | 0 | 0 | 10 |  |
| Collinsella | 3-(4-Hydroxyphenyl) propionic acid | -0.95152 | 0 | 0 | 10 |  |
| **Family** |  |  |  |  |  |  |
| Bacteroidaceae | 5-Aminovaleric acid | 0.923077 | 0 | 0 | 12 |  |
| Bacteroidaceae | Creatine | 0.902098 | 0 | 0 | 12 |  |
| Prevotellaceae | 3-(4-Hydroxyphenyl) propionic acid | 0.963224 | 4.98E-07 | 0.000274 | 12 |  |
| Prevotellaceae | NMN | 0.915245 | 2.98E-05 | 0.008726 | 12 |  |
| Prevotellaceae | N8-Acetylspermidine | 0.914187 | 3.17E-05 | 0.008726 | 12 |  |
| Prevotellaceae | N1, N8-Diacetylspermidine | 0.914187 | 3.17E-05 | 0.008726 | 12 |  |
| Bifidobacteriaceae | 5-Oxoproline | -0.93939 | 0 | 0 | 10 |  |
| Bifidobacteriaceae | Trp | -0.96364 | 0 | 0 | 10 |  |
| Coriobacteriaceae | Ser | -0.95804 | 0 | 0 | 12 |  |
| Coriobacteriaceae | Inosine | -0.94406 | 0 | 0 | 12 |  |
| Family_XIII | 2-Hydroxyvaleric acid | -0.93939 | 0 | 0 | 10 |  |
| Family_XIII | Putrescine | -0.96364 | 0 | 0 | 10 |  |
| Rikenellaceae | Homovanillic acid | -0.95622 | 1.18E-06 | 0.000258 | 12 |  |
| Rikenellaceae | Phe | -0.94221 | 4.61E-06 | 0.000882 | 12 |  |
| Family_XIII | 2-Methylserine | -0.95672 | 1.46E-05 | 0.00234 | 10 |  |
| Rikenellaceae | 3-Hydroxybutyric acid | -0.92614 | 1.53E-05 | 0.00234 | 12 |  |
| Rikenellaceae | 2-Hydroxyvaleric acid | -0.92119 | 2.09E-05 | 0.002917 | 12 |  |
| Rikenellaceae | 2-Methylserine | -0.91634 | 2.8E-05 | 0.003575 | 12 |  |
| Rikenellaceae | Putrescine | -0.91419 | 3.17E-05 | 0.003733 | 12 |  |
| Rikenellaceae | Inosine | -0.90718 | 4.63E-05 | 0.004901 | 12 |  |
| Rikenellaceae | N-Acetylputrescine | -0.90564 | 5.02E-05 | 0.004901 | 12 |  |
| Rikenellaceae | Isopropanolamine | -0.90481 | 5.24E-05 | 0.004901 | 12 |  |
| Coriobacteriaceae | 2-Methylserine | -0.90406 | 5.44E-05 | 0.004901 | 12 |  |
| Rikenellaceae | Mevalonic acid | -0.90018 | 6.59E-05 | 0.005606 | 12 |  |
| Bifidobacteriaceae | Carboxymethyllysine | -0.92402 | 0.000133 | 0.007833 | 10 |  |
| **Pylum** |  |  |  |  |  |  |
| Actinobacteria | Creatine | -0.9352 | 8.06E-06 | 0.003671 | 12 |  |
| Actinobacteria | Trp | -0.91769 | 2.59E-05 | 0.004379 | 12 |  |
| Actinobacteria | Inosine | -0.91419 | 3.17E-05 | 0.004379 | 12 |  |
| Actinobacteria | Ser | -0.91068 | 3.85E-05 | 0.004379 | 12 |  |
| Actinobacteria | Propionic acid | -0.90368 | 5.54E-05 | 0.004863 | 12 |  |
