# Supplementary material for: Half-Elemental Diet Shifts the Human Intestinal Bacterial Compositions and Metabolites: A Pilot Study with Healthy Individuals
Source: Gastroenterol Res Pract. 2020 Aug 6;2020:7086939. doi: 10.1155/2020/7086939 (PMC7428940; doi:10.1155/2020/7086939)
Supplement: Supplementary 9 — Table S7: significant correlations between bacterial taxonomy and butyric acid/propionic acid. [file 7086939.f9.docx]

Table S7. Significant correlations between bacterial taxonomy and butyric acid/propionic acid

| **Genus** | **Metabolite** | **Correlation**  **coefficient** | ***p* value** | ***q* value** | **Sample size** |
| --- | --- | --- | --- | --- | --- |
| uncultured Porphyromonadaceae | Butyric acid | 0.762 | 0.006 | 0.094 | 12 |
| [Eubacterium]_coprostanoligenes_group | Butyric acid | -0.833 | 0.008 | 0.101 | 9 |
| Streptococcus | Butyric acid | -0.643 | 0.028 | 0.159 | 12 |
| Bacteroides | Propionic acid | 0.825 | 1.7E-03 | 0.068 | 12 |
| Subdoligranulum | Propionic acid | 0.720 | 2.9E-02 | 0.162 | 9 |
| uncultured Lachnospiraceae | Propionic acid | 0.687 | 1.4E-02 | 0.124 | 12 |
| Prevotella_9 | Propionic acid | 0.663 | 1.9E-02 | 0.142 | 12 |
| Parabacteroides | Propionic acid | 0.629 | 3.2E-02 | 0.170 | 12 |
| [Eubacterium]_coprostanoligenes_group | Propionic acid | -0.883 | 3.1E-03 | 0.080 | 9 |
| Collinsella | Propionic acid | -0.818 | 6.8E-03 | 0.095 | 10 |
| Bifidobacterium | Propionic acid | -0.794 | 9.8E-03 | 0.107 | 10 |
| Ruminiclostridium_5 | Propionic acid | -0.791 | 6.1E-03 | 0.095 | 11 |
| Alistipes | Propionic acid | -0.753 | 4.7E-03 | 0.087 | 12 |
| Shuttleworthia | Propionic acid | -0.740 | 2.3E-02 | 0.153 | 9 |
| Roseburia | Propionic acid | -0.734 | 9.1E-03 | 0.105 | 12 |
| Ruminococcaceae_UCG-002 | Propionic acid | -0.688 | 1.9E-02 | 0.143 | 11 |
| Ruminococcus_1 | Propionic acid | -0.636 | 4.0E-02 | 0.186 | 11 |
| Odoribacter | Propionic acid | -0.587 | 4.9E-02 | 0.202 | 12 |
| **Family** |  |  |  |  |  |
| Streptococcaceae | Butyric acid | -0.643 | 0.028 | 0.120 | 12 |
| Bacteroidaceae | Propionic acid | 0.825 | 1.7E-03 | 0.045 | 12 |
| Prevotellaceae | Propionic acid | 0.799 | 1.8E-03 | 0.047 | 12 |
| Coriobacteriaceae | Propionic acid | -0.825 | 1.7E-03 | 0.045 | 12 |
| Bifidobacteriaceae | Propionic acid | -0.794 | 9.8E-03 | 0.077 | 10 |
| Rikenellaceae | Propionic acid | -0.753 | 4.7E-03 | 0.057 | 12 |
| Family_XIII | Propionic acid | -0.745 | 1.8E-02 | 0.103 | 10 |
| **Phylum** |  |  |  |  |  |
| Bacteroidetes | Propionic acid | 0.846 | 9.7E-04 | 0.030 | 12 |
| Actinobacteria | Propionic acid | -0.904 | 5.5E-05 | 0.010 | 12 |
