# Supplementary material for: Half-Elemental Diet Shifts the Human Intestinal Bacterial Compositions and Metabolites: A Pilot Study with Healthy Individuals
Source: Gastroenterol Res Pract. 2020 Aug 6;2020:7086939. doi: 10.1155/2020/7086939 (PMC7428940; doi:10.1155/2020/7086939)
Supplement: Supplementary 10 — Table S8: significant correlations between bacterial taxonomy and bile acids. [file 7086939.f10.docx]

Table S8. Significant correlations between bacterial taxonomy and bile acids

| **Genus** | **Metabolite** | **Correlation**  **coefficient** | ***p* value** | ***q* value** | **Sample size** |
| --- | --- | --- | --- | --- | --- |
| Prevotella_9 | Cholic acid | 0.977 | 0.000 | 0.000 | 12 |
| uncultured Lachnospiraceae | Cholic acid | 0.865 | 0.000 | 0.035 | 12 |
| Parasutterella | Cholic acid | 0.829 | 0.021 | 0.150 | 7 |
| [Ruminococcus]_gnavus_group | Cholic acid | 0.783 | 0.004 | 0.087 | 12 |
| Ruminococcus_1 | Cholic acid | -0.927 | 0.000 | 0.000 | 11 |
| Roseburia | Cholic acid | -0.909 | 0.000 | 0.000 | 12 |
| Anaerotruncus | Cholic acid | -0.85 | 0.007 | 0.098 | 8 |
| Ruminococcaceae_NK4A214_group | Cholic acid | -0.847 | 0.016 | 0.136 | 7 |
| Sellimonas | Cholic acid | -0.845 | 0.002 | 0.070 | 10 |
| Ruminococcaceae_UCG-002 | Cholic acid | -0.789 | 0.004 | 0.087 | 11 |
| uncultured Ruminococcaceae | Cholic acid | -0.733 | 0.031 | 0.167 | 9 |
| Alistipes | Cholic acid | -0.732 | 0.007 | 0.095 | 12 |
| Bifidobacterium | Cholic acid | -0.721 | 0.024 | 0.155 | 10 |
| [Ruminococcus]_gnavus_group | Glycocholic acid | 0.818 | 0.002 | 0.070 | 12 |
| Phascolarctobacterium | Glycocholic acid | 0.717 | 0.037 | 0.178 | 9 |
| Prevotella_9 | Glycocholic acid | 0.713 | 0.009 | 0.106 | 12 |
| uncultured Lachnospiraceae | Glycocholic acid | 0.669 | 0.017 | 0.136 | 12 |
| Bacteroides | Glycocholic acid | 0.650 | 0.026 | 0.158 | 12 |
| Faecalibacterium | Glycocholic acid | -0.755 | 0.007 | 0.095 | 12 |
| Roseburia | Glycocholic acid | -0.755 | 0.007 | 0.095 | 12 |
| Prevotella_9 | Taurocholic acid | 0.892 | 0.000 | 0.025 | 12 |
| uncultured Lachnospiraceae | Taurocholic acid | 0.851 | 0.000 | 0.041 | 12 |
| Parasutterella | Taurocholic acid | 0.811 | 0.027 | 0.158 | 7 |
| [Ruminococcus]_gnavus_group | Taurocholic acid | 0.741 | 0.008 | 0.101 | 12 |
| Phascolarctobacterium | Taurocholic acid | 0.733 | 0.031 | 0.167 | 9 |
| Dorea | Taurocholic acid | 0.663 | 0.019 | 0.142 | 12 |
| Bacteroides | Taurocholic acid | 0.657 | 0.024 | 0.155 | 12 |
| Ruminococcus_1 | Taurocholic acid | -0.891 | 0.000 | 0.040 | 11 |
| Barnesiella | Taurocholic acid | -0.862 | 0.003 | 0.075 | 9 |
| Alistipes | Taurocholic acid | -0.858 | 0.000 | 0.039 | 12 |
| Ruminococcaceae_UCG-002 | Taurocholic acid | -0.826 | 0.002 | 0.068 | 11 |
| Roseburia | Taurocholic acid | -0.818 | 0.002 | 0.070 | 12 |
| Bifidobacterium | Taurocholic acid | -0.782 | 0.012 | 0.117 | 10 |
| Ruminiclostridium_5 | Taurocholic acid | -0.773 | 0.008 | 0.101 | 11 |
| uncultured Ruminococcaceae | Taurocholic acid | -0.75 | 0.025 | 0.156 | 9 |
| Ruminococcus_2 | Taurocholic acid | -0.747 | 0.033 | 0.171 | 8 |
| Odoribacter | Taurocholic acid | -0.587 | 0.049 | 0.202 | 12 |
| **Family** |  |  |  |  |  |
| Lachnospiraceae | Cholic acid | 0.608 | 0.040 | 0.142 | 12 |
| Erysipelotrichaceae | Cholic acid | -0.776 | 0.005 | 0.057 | 12 |
| Ruminococcaceae | Cholic acid | -0.776 | 0.005 | 0.057 | 12 |
| Rikenellaceae | Cholic acid | -0.732 | 0.007 | 0.066 | 12 |
| Bifidobacteriaceae | Cholic acid | -0.721 | 0.024 | 0.118 | 10 |
| Desulfovibrionaceae | Cholic acid | -0.657 | 0.039 | 0.141 | 10 |
| Alcaligenaceae | Cholic acid | -0.636 | 0.03 | 0.125 | 12 |
| Bacteroidaceae | Glycocholic acid | 0.650 | 0.026 | 0.118 | 12 |
| Ruminococcaceae | Glycocholic acid | -0.769 | 0.005 | 0.061 | 12 |
| Acidaminococcaceae | Taurocholic acid | 0.717 | 0.037 | 0.138 | 9 |
| Bacteroidaceae | Taurocholic acid | 0.657 | 0.024 | 0.118 | 12 |
| Prevotellaceae | Taurocholic acid | 0.641 | 0.025 | 0.118 | 12 |
| Rikenellaceae | Taurocholic acid | -0.858 | 0.000 | 0.025 | 12 |
| Bifidobacteriaceae | Taurocholic acid | -0.782 | 0.012 | 0.084 | 10 |
| Coriobacteriaceae | Taurocholic acid | -0.727 | 0.010 | 0.077 | 12 |
| Ruminococcaceae | Taurocholic acid | -0.727 | 0.010 | 0.077 | 12 |
| Family_XIII | Taurocholic acid | -0.709 | 0.028 | 0.120 | 10 |
| Alcaligenaceae | Taurocholic acid | -0.587 | 0.049 | 0.155 | 12 |
| **Phylum** |  |  |  |  |  |
| Actinobacteria | Cholic acid | -0.630 | 0.028 | 0.127 | 12 |
| Proteobacteria | Glycocholic acid | 0.734 | 0.009 | 0.076 | 12 |
| Bacteroidetes | Glycocholic acid | 0.643 | 0.028 | 0.127 | 12 |
| Actinobacteria | Glycocholic acid | -0.620 | 0.032 | 0.135 | 12 |
| Actinobacteria | Taurocholic acid | -0.757 | 0.004 | 0.062 | 12 |
